# Distinct trajectory of gut microbiota driven by a human oral commensal: insights from a murine study

**DOI:** 10.1080/20002297.2025.2569524

**Published:** 2025-10-24

**Authors:** Wei-Ting Lin, Shiao-Pieng Lee, Chin Li, Chia-Bin Chang, Hsiu-Chuan Chien, Jann-Tay Wang, Song-Chou Hsieh, Shu-Fen Wu, Yu-Chao Tseng

**Affiliations:** a Division of Oral and Maxillofacial Surgery, Department of Dentistry, Ditmanson Medical Foundation Chia-Yi Christian Hospital, Chiayi, Taiwan; b Division of Oral and Maxillofacial Surgery, Department of Dentistry, Tri-Service General Hospital, Taipei, Taiwan; c School of Dentistry, National Defense Medical University, Taipei, Taiwan; d Department of Biomedical Sciences, Institute of Molecular Biology, and Institute of Biomedical Sciences, National Chung Cheng University, Chiayi, Taiwan; e Department of Urology, Ditmanson Medical Foundation Chia-Yi Christian Hospital, Chiayi, Taiwan; f Department of Laboratory Medicine, Ditmanson Medical Foundation Chia-Yi Christian Hospital, Chiayi, Taiwan; g Department of Internal Medicine, National Taiwan University Hospital, Taipei, Taiwan; h Department of Biomedical Sciences, Epigenomics and Human Disease Research Center, National Chung Cheng University, Chiayi, Taiwan; i Department of Internal Medicine, Ditmanson Medical Foundation Chia-Yi Christian Hospital, Chiayi, Taiwan; j Department of Medical Research, Ditmanson Medical Foundation Chia-Yi Christian Hospital, Chiayi, Taiwan

**Keywords:** Oral-gut axis, *Haemophilus parainfluenzae*, *Bacteroides acidifaciens*, ectopic colonization, postbiotic, immunomodulation, post-antibiotic reconstitution, NOD mice

## Abstract

**Background:**

Oral microbes modulate the gut microbiota. *Haemophilus parainfluenzae*, a core human oral commensal with immunomodulatory properties, is reduced in autoimmune diseases, while mitigating Sjögren's syndrome-like disease with improved oral microbiota in female NOD mice. However, whether it modulates the gut microbiota remains unknown.

**Objective:**

To study the modulatory effect of oral *H. parainfluenzae* inoculation on the gut microbiota.

**Design:**

Female NOD mice were orally inoculated with *H. parainfluenzae* following antibiotic treatment. Fecal samples were collected pre- and post-inoculation for 16S rRNA gene sequencing. Splenic antigen-presenting cells were analyzed for systemic immunomodulation.

**Results:**

Despite prominent convergence of diversity and beta dissimilarity within each group, *H. parainfluenzae* led to distinct core microbiota and overall microbial community. While reducing the Firmicutes-to-Bacteroidetes ratio, *H. parainfluenzae* enriched Bacteroidaceae and its genus *Bacteroides*. *Bacteroides acidifaciens*, a beneficial gut commensal, was enriched in ASV-level analyses. The splenic dendritic cells were reduced. Notably, neither did *H. parainfluenzae* establish ectopic gut colonization, nor was sustained oral colonization required, indicating that non-viable microbes may be sufficient to direct these responses.

**Conclusions:**

*H. parainfluenzae* drives a distinct gut microbiota reconstitution trajectory, characterized by *B. acidifaciens* enrichment without establishing notable colonizations, supporting its role in the oral-gut axis and warranting future postbiotic research.

## KEY MESSAGES

The beneficial human oral commensal *Haemophilus parainfluenzae* drives a distinct post-antibiotic reconstitution trajectory of the gut microbiota in female NOD mice.

The treatment enriched *Bacteroides acidifaciens*, a gut commensal with multifaceted benefits, while a decrease in splenic CD11c^+^CD11b^−^ cells (representative of dendritic cells) suggests systemic immunomodulation.

*H. parainfluenzae* inoculation did not establish ectopic gut colonization, and sustained oral colonization was not required for these effects, indicating that non-viable microbes may be sufficient to direct these responses, supporting future investigation of *H. parainfluenzae* as a postbiotic.

## Introduction

The gut microbiota comprises a vast and diverse microbial community that support key physiological processes in the host. While its balance benefits overall host health, disruption of this balance, or dysbiosis, results in a wide range of diseases [1]. Thus, it is crucial to understand the factors that shape the gut microbiota and the mechanisms through which it recovers from disturbances, such as antibiotic exposure.

Several factors help define the gut microbiota. Host-derived factors, such as immune responses and gastrointestinal secretions, shape the microbial landscape, while dietary components enrich specific bacterial groups [2,3]. Environmental factors, including delivery mode and social interactions, facilitate microbiota transmission [4,5]. Microbial interspecies interactions, such as resource competition and antimicrobial compound production, help maintain microbial balance [6]. Dysbiosis may occur during disruptions of these regulatory mechanisms.

The oral microbiota is the second-largest microbial community after the gut [7]. As a source of gut microbes, it modulates the gut microbiota through the continuous introduction of oral microorganisms and their products [8,9]. Periodontal pathogens such as *Porphyromonas gingivalis*, *Fusobacterium nucleatum*, and *Aggregatibacter actinomycetemcomitans* contribute to gut dysbiosis, potentially exacerbating inflammation [10]. Normal oral microbiota such as *Streptococcus mitis* and *Streptococcus salivarius* also exhibit modulatory effects [10]. Beyond individual microbes, studies have highlighted systemic modulatory effects of oral interventions. Nonsurgical mechanical debridement with or without chlorhexidine reduced peri-implant mucositis associated with decreased oral pathobionts in a clinical trial [11], while a meta-analysis reported potential benefits of nonsurgical periodontal treatment on carotid intima-media thickness and flow-mediated dilatation in patients with periodontitis [12]. These findings suggest that modulation of the oral microbiota—whether through antiseptics or by providing individual bacterial strains—may carry systemic and gut-related consequences. Nevertheless, much remains unknown about the broader oral microbiota, particularly the contribution of beneficial oral microbes to gut health.

*Haemophilus parainfluenzae* is a beneficial oral commensal bacterium with immunomodulatory properties [13,14]. Its oral depletion has been associated with autoimmune and chronic inflammatory diseases [13–17]. Using a commonly adopted mouse model, we recently showed that *H. parainfluenzae* restored immune homeostasis, ameliorated Sjögren's syndrome-like disease, and enhanced oral microbial diversity and resilience in female NOD mice [14]. To test whether *H. parainfluenzae* exerts a modulatory effect on the gut microbiota, similar to periodontal pathogens or other oral commensals, the present study investigated the effects of oral inoculation on the gut microbiota and splenic antigen-presenting cells during post-antibiotic microbial reconstitution in female NOD mice. This study extends our prior report and demonstrates that *H. parainfluenzae* induces distinct shifts in the gut microbiota, characterized by enrichment of the beneficial gut microbe *Bacteroides acidifaciens* and a reduction in splenic dendritic cells. This work contributes to a deeper understanding of the oral-gut axis.

## Methods

### Animal and experimental design

**Figure 1.**
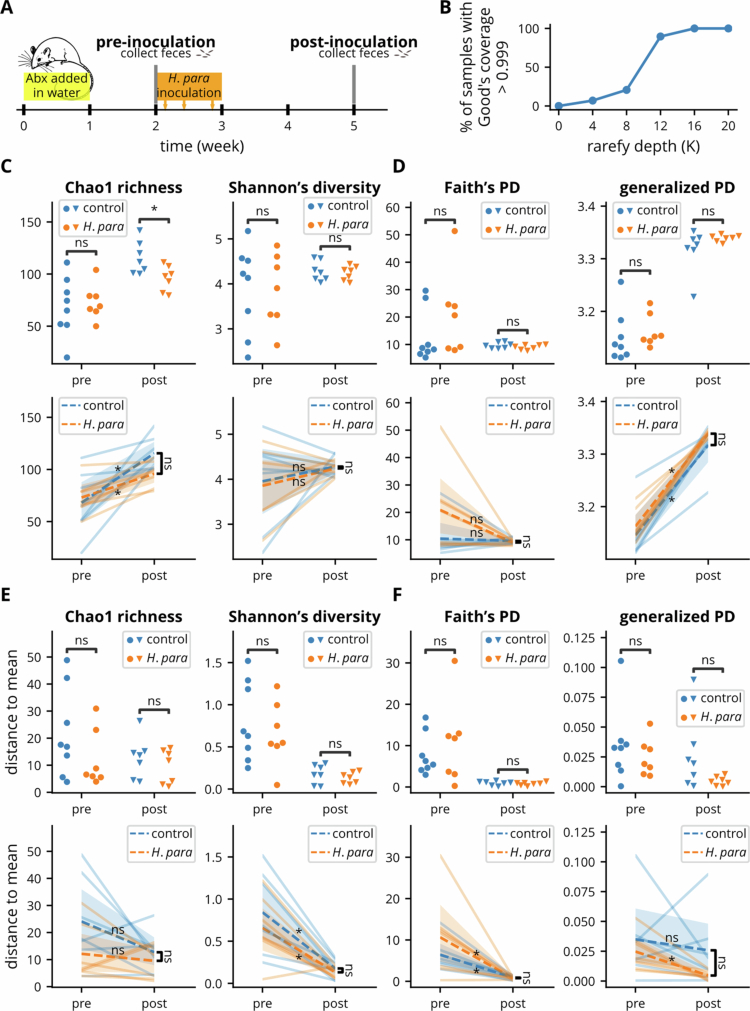
Overview of experimental design and microbial alpha-diversity metrics. (A) Experimental timeline. (B) Rarefaction curve showing the percentage of samples with adequate sequencing depth, defined as Good’s coverage > 0.999. (C–D) Alpha diversity metrics comparing control and *H. parainfluenzae* groups before and after inoculation. Paired analyses are displayed in the lower rows. Non-phylogenetic (C) and phylogenetic richness and diversity (D). (E–F) Distance-to-mean analyses illustrating individual variation within each group. Paired analyses are displayed in the lower rows. Non-phylogenetic (E) and phylogenetic richness and diversity (F). Statistical comparisons were performed using the Wilcoxon signed-rank test for paired analyses and the Mann-Whitney U test for between-group comparisons. Statistical significance: *p* < 0.05 (*) and non-significant (ns).

NOD/ShiLtJNarl mice obtained from the National Laboratory Animal Center (Taipei, Taiwan) were housed under specific pathogen-free conditions. To ensure more precise interpretation, the experimental design closely followed our previous report (Figure 1A) [14]. Six-week-old female NOD mice were given an antibiotic cocktail consisting of 0.5 mg/ml ampicillin (Sigma-Aldrich), 0.5 mg/ml gentamicin (Acros Organics), and 0.25 mg/ml vancomycin (Sigma-Aldrich) in their drinking water for one week. After a one-week washout period to minimize residual antibiotic effects that could confound colonization, mice were randomly assigned to either the control or *H. parainfluenzae* group using an alternating sequence.

The sample size was estimated from the relative abundance table in a previous study investigating murine gut microbiota following administration of *A. actinomycetemcomitans* [18]. A sample size of six mice per group was sufficient to detect high-ranking differential species (e.g. *Turicibacter* sp. in the study) with 80% power at an *α* level of 0.05. The final sample sizes were eight mice in the control group and seven in the *H. parainfluenzae* group, both to improve the detection threshold and to account for animal availability at the time of the experiment. One mouse in the control group failed to provide a fecal sample at the post-inoculation time point and was excluded from paired analysis.

The treatment group received three doses of *H. parainfluenzae* (acquired from the National Taiwan University Hospital, Taipei, Taiwan) with each dose containing 2 × 10^6^ CFU in a 20 μl bacterial suspension retained in the mouth floor for 30 min under anesthesia at prone position. The dose was scaled according to the estimated 1,000-fold size difference between the oral and gut microbiota, aligning with gut study doses typically ranging from 10^8^ to 10^10^ CFU [7,19]. The *H. parainfluenzae* preparation was validated using the VITEK 2 and Phoenix automated identification systems and by qPCR with an *H. parainfluenzae*–specific probe [15]. Fecal samples were collected at baseline and two weeks after inoculation. To minimize confounders, samples would be excluded if the mice became hyperglycemic. Approval was granted by the Institutional Animal Care and Use Committee of National Chung Cheng University (1110907).

### Fecal DNA extraction and 16S rRNA gene sequencing

Fecal samples were resuspended in PBS, centrifuged to remove debris, and washed twice with PBS. DNA was extracted using the QIAamp DNA Stool Mini Kit (Qiagen). The V3-V4 regions of the bacterial 16S rRNA gene were amplified from purified DNA. A second-stage PCR using the Nextera XT index kit (Illumina) was performed to generate sequencing-ready libraries. Sequencing was conducted on the Illumina MiSeq platform with 18 dark cycles and 350 read cycles for the forward read, and 18 dark cycles and 250 read cycles for the reverse read.

### Microbiota analysis

Further microbiota analyses were performed in QIIME2 (v2023.2), unless otherwise indicated [20]. Amplicon sequence variants (ASVs) were generated following denoising and merging using DADA2 [21]. The phylogenetic tree was constructed following the align-to-tree-mafft-iqtree pipeline [22,23]. Further analysis was performed after rarefaction at 16,000 reads per sample, with adequate sequencing depth confirmed by Good’s coverage.

Alpha diversity indices (Chao1, Pielou’s evenness, Shannon, and Faith’s PD) were calculated with QIIME2, while generalized Faith’s PD was computed with the scikit-bio package (v0.6.2) in Python. The core ASV was defined as being present in at least half of the samples in the subgroup. Bray–Curtis and weighted and unweighted UniFrac distances were used to compute dissimilarity; principal coordinate analysis (PCoA) was applied to visualize microbial community structure, with PERMANOVA used to test for group differences. Distance-to-centroid analysis was performed to evaluate within-group dispersion.

Taxon annotation was performed using the classify-consensus-vsearch pipeline based on VSEARCH against the SILVA database (v138) [24,25]. To improve species-level resolution, ASVs were submitted to the NCBI website (https://blast.ncbi.nlm.nih.gov/) using BLAST against the NCBI database (core_nt) with results filtered by annotated taxa from the previous step. Relative abundance was used for data visualization.

For analysis at the phylum level, phyla with a mean abundance of less than 0.1% were excluded. The log-transformed abundance ratio of key phyla was compared between groups using the Student's *t*-test. For analysis at lower levels, taxa with a median abundance of zero in target analysis groups were excluded to minimize background noise.

ANCOM-BC and ANCOM-BC2 were applied to identify differentially abundant taxa [26,27]. ANCOM-BC and paired ANCOM-BC2 analyses were performed using the ANCOMBC package (v2.8.1) in R (v4.4.2), respectively, with Benjamini-Hochberg correction to control the false discovery rate at *q* < 0.15.

### Flow cytometric analysis of splenic antigen-presenting cells

Mice were euthanized during the post-inoculation period. Spleens were harvested and processed into single-cell suspensions. After lysing red blood cells, cells were stained at 4 °C for 30 min in the dark with the following fluorescent dye-conjugated antibodies: anti-mouse CD45 (30-F11), anti-mouse CD11c (HL3), anti-mouse CD80 (16-10A1), anti-mouse CD86 (GL1) (BD Biosciences), and anti-mouse CD11b (M1−70) (BioLegend).

Surface marker expression was evaluated using a FACSAria III flow cytometer (BD Biosciences). Data were analyzed with FlowJo software (BD Biosciences). The gating strategy sequentially selected mononuclear cells, followed by single cells, and finally CD45^+^ cells. Antigen-presenting cell (APC) subsets were defined according to CD11b and CD11c expression. Co-stimulatory molecule expression (CD80 and CD86) was quantified within APC subsets. Statistical comparisons were performed using Student's *t*-test.

### Assessment of fecal and salivary presence of *H. parainfluenzae*

To determine whether colonization of *H. parainfluenzae* occurred in the gut, ASVs were compared against available 16S sequences of *H. parainfluenzae* in the NCBI database using VSEARCH. Saliva samples were collected from mice at pre- and post-inoculation periods under anesthesia after intraperitoneal administration of 0.225 mg/kg pilocarpine hydrochloride (Sigma-Aldrich). Salivary ASVs were generated and analyzed for the presence of *H. parainfluenzae* following the same process.

### Statistical analysis

Paired and unpaired comparisons were conducted using the Wilcoxon signed-rank test and Mann-Whitney U test, respectively, unless stated otherwise. Statistical analyses were performed using the Python packages statannotations (v0.6) and SciPy (v1.10.1). Confidence interval estimates were calculated using the seaborn package (v0.12.2) with the bootstrap method. Statistical significance was set at *p* < 0.05.

## Results

### Microbial alpha-diversity changes

To assess the impact of *H. parainfluenzae* on gut microbiota reconstitution, antibiotics were administered via drinking water prior to inoculation, followed by fecal sample collection at pre- and post-inoculation time points (Figure 1A). Mice from both groups remained euglycemic at the post-inoculation time point. Rarefaction curves of Good's coverage confirmed sufficient sequencing depth, with 16,000 reads used for further analysis (Figure 1B). Comparison of alpha diversity metrics revealed higher Chao1 richness in the control group but no other metrics post-inoculation (Figure 1C, supplementary Figure 1A). This non-significant trend persisted when considering phylogenetic diversity, including Faith’s PD and its generalized version (Figure 1D), suggesting that the increased richness in the control group may be due to differences in phylogenetically close ASVs.

For paired analysis (Figure 1C–D, Supplementary Figure 1A), Chao1 richness increased in both groups with diminished effect on Faith’s PD. Since Faith’s PD represents the sum of the total branch length of observed species, an increase in non-phylogenetic richness without a corresponding change in Faith’s PD suggests that post-inoculation ASVs were more phylogenetically related. In contrast, the generalized Faith’s PD, which accounts for species abundance, showed an increasing trend, indicating an increase in abundant species that were less phylogenetically related. Despite these changes, between-group differences were not significant.

Although evenness and diversity remained unchanged, these metrics, along with Faith's PD, converged post-inoculation (Figure 1C–D, Supplementary Figure 1A). A significant reduction in the distance-to-mean (Figure 1E–F, Supplementary Figure 1B) confirmed reduced intra-group variability for these metrics, with a notable reduction in the distance-to-mean of generalized Faith's PD observed only in the *H. parainfluenzae* group. These observations suggest that the gut microbiota in both groups reconstituted toward a steady state, although certain differences driven by abundant ASVs may persist.

### Beta-diversity analyses of microbial community structure

**Figure 2.**
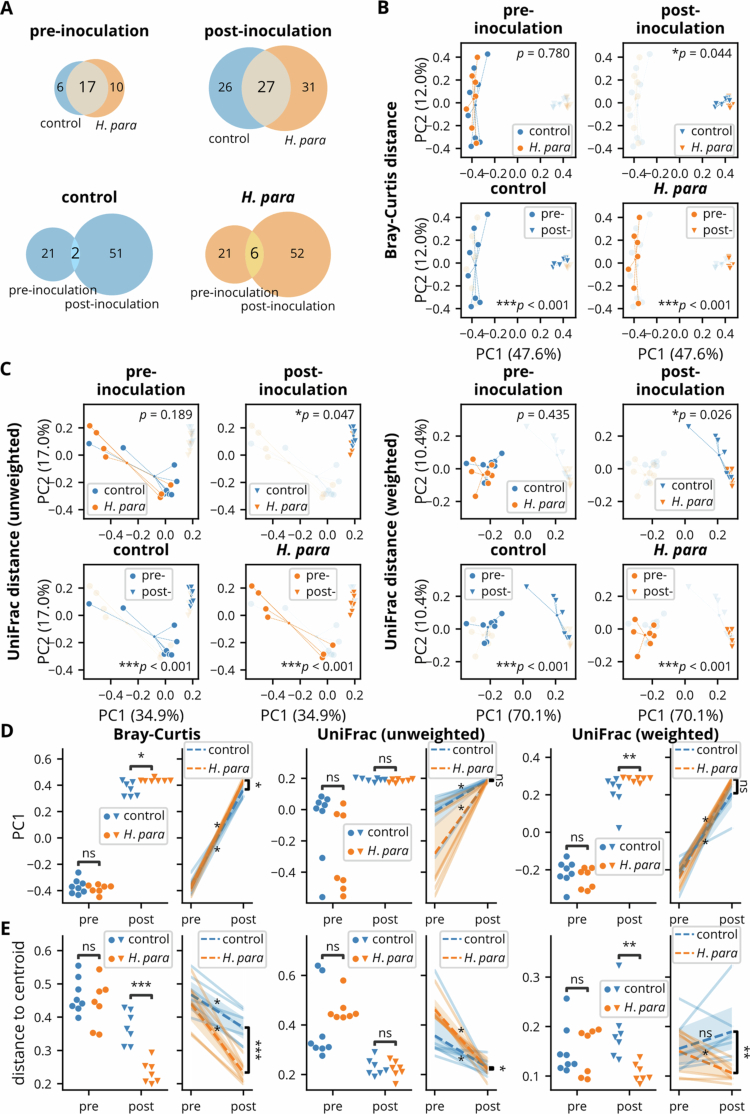
Beta-diversity analyses of microbial community structure. (A) Venn diagrams showing the number of shared and unique core amplicon sequence variants (ASVs) between the control and *H. parainfluenzae* (*H. para*) groups before and after inoculation. (B and C) Principal coordinate analysis (PCoA) based on Bray-Curtis, unweighted UniFrac, and weighted UniFrac distances, illustrating microbial community structure and differences before and after *H. parainfluenzae* inoculation in the control and *H. parainfluenzae* groups. (D) PC1 values from Bray-Curtis, unweighted UniFrac, and weighted UniFrac distances, comparingthe control and *H. parainfluenzae* groups across pre- and post-inoculation time points. (E) Distance to centroid analysis for Bray-Curtis, unweighted UniFrac, and weighted UniFracdistances, reflecting within-group dispersion of microbial communities before and after *H. parainfluenzae* inoculation. Statistical comparisons were performed using permutational multivariate analysis of variance (PERMANOVA) for beta diversity comparisons, the Wilcoxon signed-rank test for paired analyses, and the Mann-Whitney U test for between-group comparisons. Statistical significance: *p* < 0.05 (*)*, p* < 0.01 (**), *p* < 0.001 (***), and non-significant (ns).

For microbial community structure, Venn diagrams depicting core ASVs showed an overall increase in the post-inoculation period (Figure 2A, lower row), consistent with increasing richness in both groups. The minimal overlap of core ASVs between the pre- and post-inoculation phases in either group suggests substantial changes between periods. A significant number of distinct core ASVs were observed between groups post-inoculation (Figure 2A, upper right), indicating substantial differences in their microbial structures.

Principal coordinate analysis (PCoA) based on Bray-Curtis distance (Figure 2B) confirmed a significant between-group change. A substantial shift in microbial composition was observed within both groups pre- and post-inoculation (Figure 2B). These trends remained consistent when incorporating phylogenetic relationships using unweighted or weighted UniFrac distances (Figure 2C). Analysis of PC1 showed greater PC1 values in Bray-Curtis and weighted UniFrac distances (Figure 2D). In paired analysis, PC1 values increased in both groups, with divergence in PC1 changes between groups observed in Bray-Curtis distance, indicating distinct shifts in microbial community composition.

Since apparent clustering occurred in PCoA post-inoculation (Figure 2C–D), especially in the *H. parainfluenzae* group, distance-to-centroid analysis exhibited lower intra-group variation in the *H. parainfluenzae* group regarding Bray-Curtis and weighted UniFrac distances (Figure 2E). The desynchronization of this change in unweighted UniFrac distance suggested that the effect was related to abundant ASVs. In paired analysis, a reduction in intra-group variation was consistently observed in the *H. parainfluenzae* group (Figure 2E). In summary, *H. parainfluenzae* inoculation induced distinct shifts in microbial community structure, characterized by greater within-group similarity.

### Taxonomic composition shifts at the phylum level

**Figure 3.**
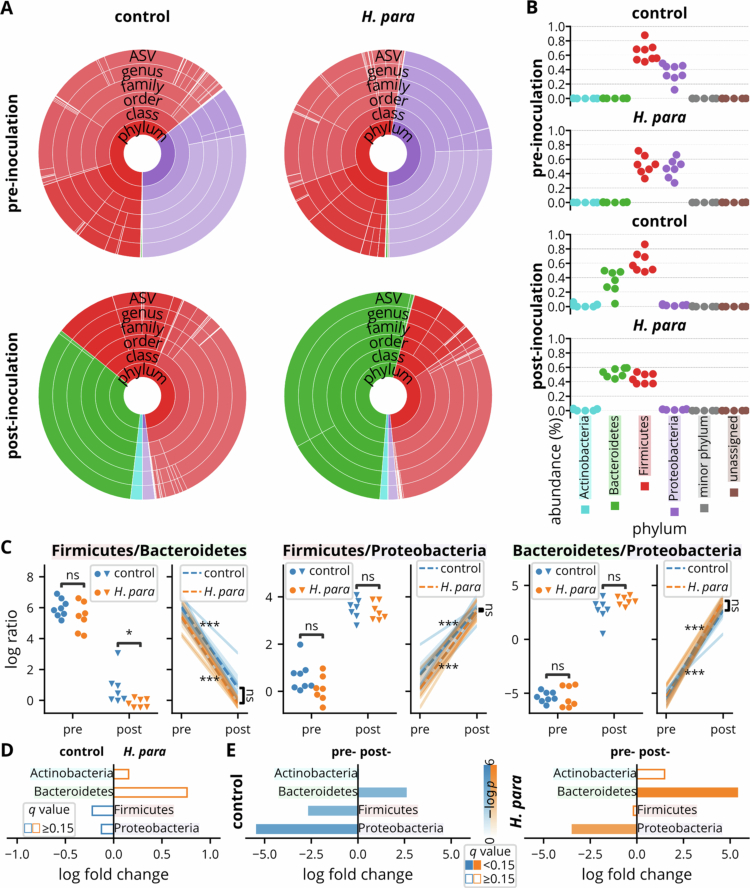
Taxonomic composition and analyses at phylum level. (A) Taxonomic composition of microbial communities across taxonomic levels in the control and *H. parainfluenzae* (*H. para*) groups before and after inoculation. The pie charts depict the mean relative abundance at each taxonomic level, with each color segment of varying depth representing an individual taxon. Colors correspond to the phyla shown in (B), indicating the phylum a taxon belongs to. (B) Relative abundance of major bacterial phyla in the control and *H. parainfluenzae* groups before and after inoculation. Different colors represent different phyla. (C) Log-transformed ratios of combinations among Firmicutes, Bacteroidetes, and Proteobacteriabefore and after inoculation in the control and *H. parainfluenzae* groups. (D) Log-fold change of major bacterial phyla (Actinobacteria, Bacteroidetes, Firmicutes, and Proteobacteria) between groups. Bars indicate the magnitude of change, with significant changes highlighted. (E) Log-fold change comparing changes pre- and post-inoculation within the control or *H. parainfluenzae* group of major bacterial phyla, with significant changes indicated. Statistical comparisons were performed using the *t*-test for log-transformed ratios (C) and the ANCOM-BC and ANCOM-BC2 methods for log fold changes (D and E). Statistical significance: *p* < 0.05 (*)*, p* < 0.01 (**), *p* < 0.001 (***), and non-significant (ns).

To assess taxonomic changes, bacterial community structures were analyzed across taxonomic levels. The major phyla included Actinobacteria, Bacteroidetes, Firmicutes, and Proteobacteria (Figure 3A–B), with log-transformed ratios showing a reduced Firmicutes-to-Bacteroidota ratio (Figure 3C). Although the Firmicutes-to-Proteobacteria ratio appeared lower in the *H. parainfluenzae* group pre-inoculation (Figure 3A–B), this difference was not statistically significant (Figure 3C). The prominent between-period changes observed in the paired analysis of both groups were consistent with the beta diversity results (Figure 3C). ANCOM-BC and ANCOM-BC2, designed for detecting compositional differences in microbiome data, revealed no significant differences between groups (Figure 3D). In the paired analysis, both groups exhibited an increase in Bacteroidetes and a decrease in Proteobacteria post-inoculation (Figure 3E).

### Differential taxa analysis across taxonomic levels

**Figure 4.**
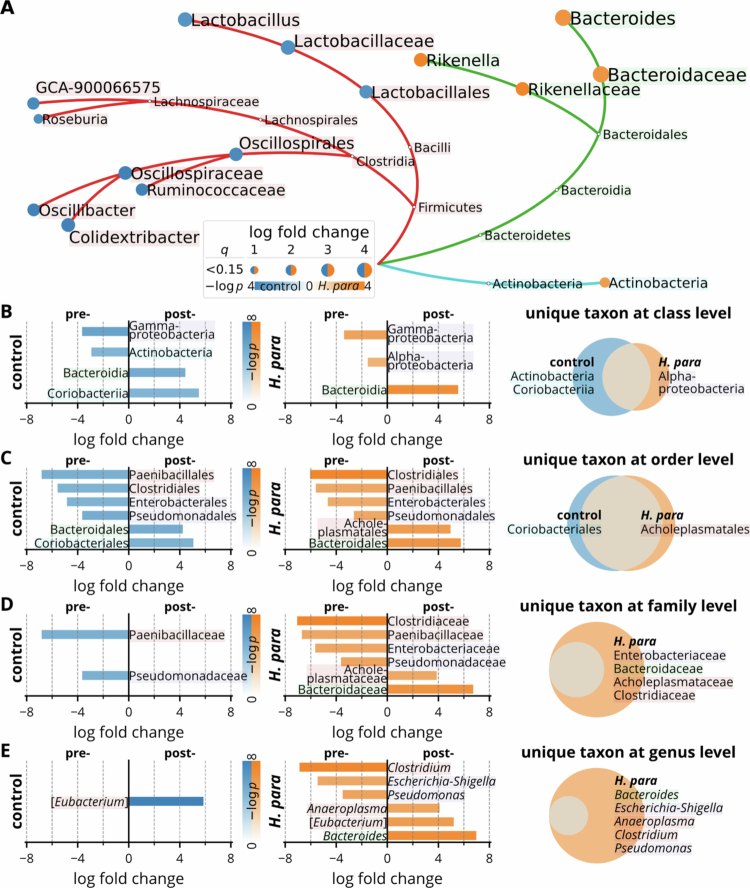
Differential abundant taxa from class to genus. (A) Phylogenetic tree highlighting differentially abundant taxa between the *H. parainfluenzae* treated and control groups. Nodes are colored based on log fold change, and significant taxa are labeled. (B–E) Log fold change of bacterial taxa at different taxonomic levels comparing pre- and postinoculation within individual groups: (B) class, (C) order, (D) family, and (E) genus. Venn’s diagrams indicate unique taxa identified in the *H. parainfluenzae* and control groups at each level. Statistical comparisons were performed using the ANCOM-BC and ANCOM-BC2 methods for log fold changes between and within groups.

Further analysis using ANCOM-BC across taxonomic levels was visualized with a taxonomic tree (Figure 4A). Taxa in Firmicutes were enriched in controls, while taxa in Bacteroidetes and Actinobacteria shifted toward the *H. parainfluenzae* group. *Bacteroides* exhibited the highest log-fold change favoring *H. parainfluenzae*.

Paired analyses, focusing on uniquely enriched or depleted taxa (Figure 4B–E), showed selective depletion of Actinobacteria in the control group at the class level (Figure 4B), consistent with their enrichment in the *H. parainfluenzae* group in the non-paired analysis (Figure 4A). At the order level, most taxa exhibited synchronized abundance changes across groups (Figure 4C). *H. parainfluenzae* selectively enriched Bacteroidaceae and Acholeplasmataceae at the family level (Figure 4D), along with their corresponding genera *Bacteroides* and *Anaeroplasma* (Figure 4E). In other hand, it selectively depleted Clostridiaceae and Enterobacteriaceae at the family level (Figure 4D), as well as *Clostridium*, *Escherichia-Shigella*, and *Pseudomonas* at the genus level (Figure 4E).

Notably, the selective enrichment of family Bacteroidaceae and genus *Bacteroides* in the *H. parainfluenzae* group aligns with the non-paired analysis while taxa in Oscillospirales, Lachnospiraceae, and Lactobacillales were not (Figure 4A, C–E). Overall, these findings demonstrate that *H. parainfluenzae* induces significant taxonomic shifts, with the family Bacteroidaceae and its genus *Bacteroides* consistently showing substantial log-fold changes.

### ASV-level taxonomic shifts

**Figure 5.**
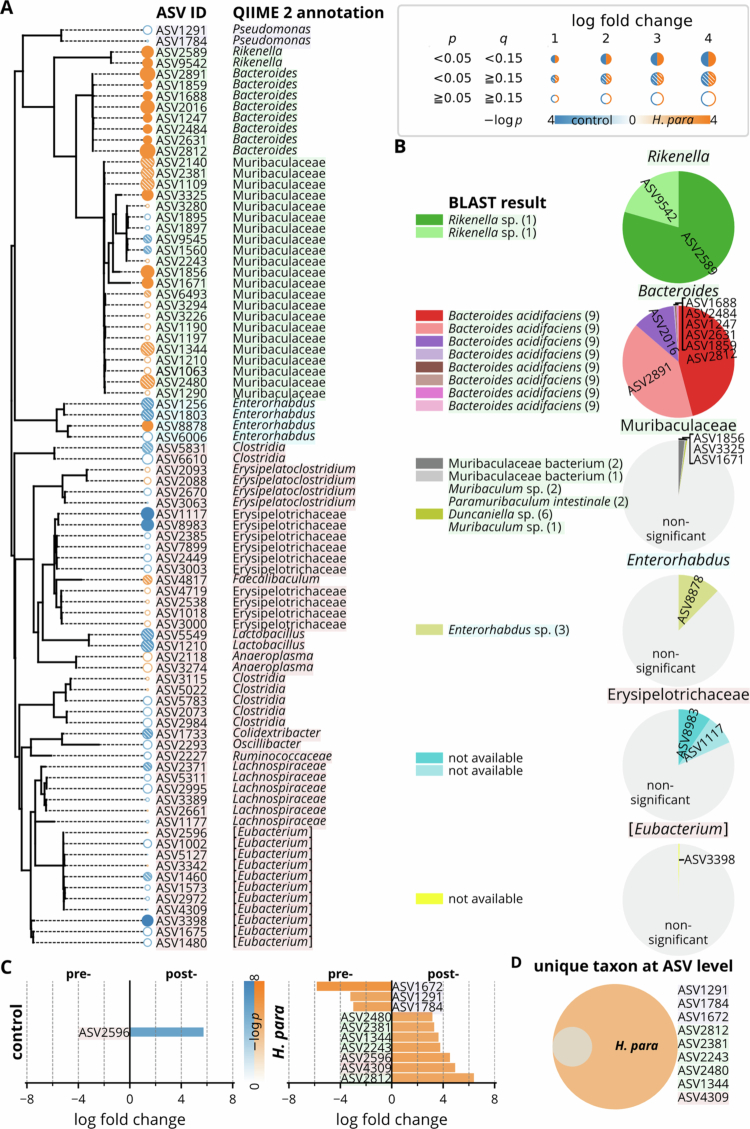
Differentially abundant ASVs. (A) Phylogenetic tree of ASVs with their corresponding taxonomic annotations, with statistical significance and log-fold changes highlighted. (B) BLAST-based taxonomic classification against the NCBI database of differentially abundant ASVs, with pie charts showing their relative abundances. Numbers in parentheses indicate the number of hits in the reference database. (C) Log fold changes of significantly altered ASVs in the control and *H. parainfluenzae* (*H. para*) groups before and after inoculation. (D) Unique ASVs identified. Statistical comparisons were performed using the ANCOM-BC and ANCOM-BC2 methods for logfold changes between and within groups.

The phylogenetic tree highlights ASVs with significant log-fold changes, showing enrichment in the *H. parainfluenzae* group, mainly *Bacteroides*, *Rikenella*, Muribaculaceae, and *Enterorhabdus* (Figure 5A). In contrast, ASVs in Erysipelotrichaceae and *Eubacterium* were enriched in the control group. All *Bacteroides* and *Rikenella* ASVs were significantly enriched in the *H. parainfluenzae* group, while ASVs in Muribaculaceae, *Enterorhabdus*, Erysipelotrichaceae, and *Eubacterium* showed variable enrichment without consistent significance. Notably, apart from *Bacteroides* and *Rikenella*, most differentially abundant taxa at higher levels (e.g. *Lactobacillus*) did not exhibit ASV-level significance (Figures 4A and 5A).

Since the SILVA database lacks species-level resolution, we annotated differentially abundant ASVs against the NCBI database. Most ASVs did not match well-established species, except for those annotated to *Bacteroides*, consistently assigned to *B. acidifaciens* (Figure 5B). Paired analysis revealed unique depletion of ASVs annotated to *Escherichia-Shigella* and *Pseudomonas* in the *H. parainfluenzae* group, along with increased abundance of ASVs assigned to *Bacteroides*, Muribaculaceae, and *Anaeroplasma* (Figure 5A, C–D). Notably, ASV2812, annotated as *Bacteroides* or *B. acidifaciens* (Figure 5A–B), was uniquely upregulated in the *H. parainfluenzae* group, exhibiting the greatest log-fold change and comprising most *Bacteroides* abundance (Figure 5B–D). These results highlight *H. parainfluenzae* inoculation in promoting gut *B. acidifaciens*.

### Alteration of splenic dendritic cell abundance by *H. parainfluenzae*

**Figure 6.**
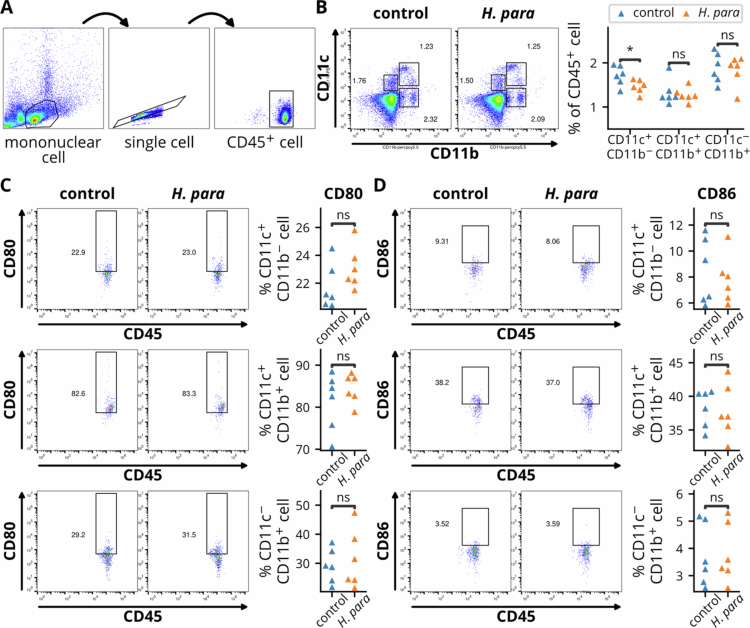
Flow cytometric analysis of splenic antigen-presenting cell (APC) subsets and costimulatory molecule expression. (A) Gating strategy for identifying CD45⁺ mononuclear cells from splenic single-cell suspensions. (B). Frequency of CD11c⁺ and CD11b⁺ cells. Left: Representative dot plots. Right panel: Quantification of APC subset frequencies within the CD45⁺ population. Subsets are defined as CD11c⁺CD11b⁺, CD11c⁺CD11b⁻, and CD11c⁻CD11b⁺. (C-D) Expression of co-stimulatory molecules CD80 and CD86 within the three APC subsets as defined in (B). Left: Representative histograms; Right: Quantification of CD80⁺ and CD86⁺ frequencies in each subset. Statistical comparisons between control and *H. parainfluenzae* (*H. para*) groups were performed using unpaired t-tests. *p* < 0.05 (*), ns: not significant.

Given the connection between gut microbiota and systemic immune responses, splenic APCs was assessed with flow cytometry (Figure 6). *H. parainfluenzae* reduced CD11c^+^CD11b^−^ cells (representative of dendritic cells), with no changes observed in the other subsets (Figure 6B). Co-stimulatory molecules CD80 and CD86 remained unchanged across all subsets (Figure 6C–D). These results indicate that *B. acidifaciens* enrichment may selectively modulate splenic dendritic cell abundance without altering their activation phenotype, suggesting decreased trafficking of dendritic cells to the spleen for antigen presentation.

### Gut and oral colonization of *H. parainfluenzae*

**Figure 7.**
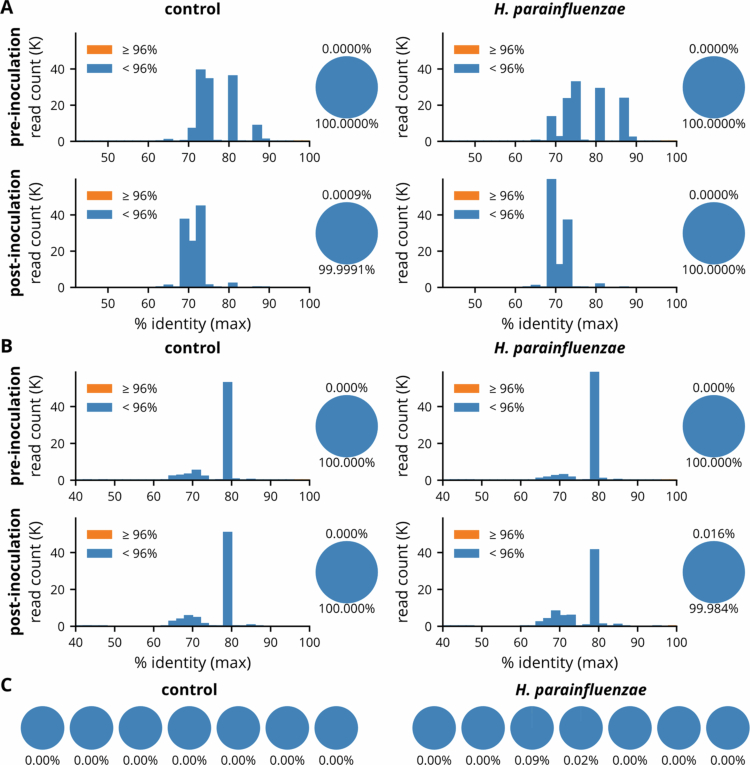
Detection of *H. parainfluenzae* in fecal and salivary microbiota. (A–B) Histograms and pie charts show the distribution and proportion of sequences based on their best-hit percent identity to reference 16S rRNA gene sequences of *H. parainfluenzae*. Each histogram displays the number of reads (in thousands, K) within each percent identity bin. Colors distinguish reads with percent identity <96% (blue) and ≥96% (orange). Adjacent pie charts summarize the proportions of high- and low-identity reads. (A) Fecal samples. (B) Salivary samples. (C) Summary of the proportion of high-identity reads (≥96%) in individual post-inoculation salivary samples in (B). Each pie represents a sample; the percentage displayed beneath each circle indicates the relative abundance of reads with ≥96% identity to *H. parainfluenzae*.

To assess whether *H. parainfluenzae* required ectopic colonization to exert its modulatory effect, ASVs were aligned with *H. parainfluenzae* reference 16S sequences (containing three near-full length 16S ribosomal genes, supplementary file) and revealed an almost complete absence in the gut (Figure 7A). In the oral cavity, *H. parainfluenzae* was undetectable pre-inoculation and detected only in a small subset of mice at very low abundance post-inoculation (Figure 7B–C). These results indicate that sustained colonization in either the oral cavity or gut is not necessary for *H. parainfluenzae* to modulate the gut microbiota.

## Discussion

This study investigated the impact of *H. parainfluenzae* on post-antibiotic reconstitution of the gut microbiota in female NOD mice, revealing a significant shift characterized by an increase in *B. acidifaciens* and a reduction in splenic dendritic cells, without notable colonization at either the gut or the oral site. These changes indicate a potential role for *H. parainfluenzae* in shaping gut microbiota.

While gut communities tend to re-establish stability post-antibiotic [28], this study demonstrated prominent reconstitution with notable microbial similarity. In contrast, our earlier study on the oral microbiota showed limited reconstitution [14], reinforcing reports of greater resilience of the oral microbiota to antibiotic-induced disruption [29]. Consequently, the oral microbiota may serve as a reservoir for gut microbiota restoration. Proteobacteria, constituting most of the salivary microbiota [14], may invade the gut, contributing to significant post-antibiotic abundance. The unique reduction of *Pseudomonas* in the *H. parainfluenzae* group also corresponds to the decline in oral *Pseudomonas mendocina* [14], highlighting a critical role of the oral microbiota in shaping gut microbiota.

Periodontitis is one of the best-studied examples illustrating the oral–gut axis. Saliva from periodontitis patients or selected periodontal pathogens alters the gut microbiota and promotes inflammation in mice [10], while patients with periodontitis are consistently associated with gut dysbiosis [30–35]. Periodontitis, with oral dysbiosis, may contribute to oxidative stress and systemic inflammation, which can be amplified by gut dysbiosis [36,37]. Interestingly, oral *H. parainfluenzae* is also reduced in periodontitis patients, while it inhibits the adhesion of *P. gingivalis*, a keystone periodontal pathogen, in vitro [17,38]. Moreover, *H. parainfluenzae*, as a nitrate-reducing bacterium, converts nitrate to nitrite, providing a direct substrate for nitric oxide (NO) production [39,40], which in turn helps maintain redox balance and limit oxidative injury [41,42]. Further studies targeting oxidative stress in relation to *H. parainfluenzae*, with particular emphasis on nitrogen metabolism and associated changes in the gut microbial community, would provide more in-depth insights.

*H. parainfluenzae* promotes the enrichment of *B. acidifaciens*, a gut commensal increasingly recognized for its multifaceted health benefits. Evidence from animal studies highlights its role in maintaining gut and liver health, correcting metabolic derangements, and enhancing mucosal immunity [43–49]. The present study further suggests potential modulation of systemic immune responses through reducing splenic dendritic cell abundance.

There is also growing evidence from human studies using a qPCR-based approach indicating depletion of gut *B. acidifaciens* in alcoholic liver disease and ulcerative colitis [48,49]. Although human oral microbiota studies focusing on alcoholic liver disease are still lacking, the observed depletion of oral *Haemophilus* or *H. parainfluenzae* in patients of Crohn’s disease and ulcerative colitis provides valuable insight into the translational relevance of the current findings [15]. Overall, *H. parainfluenzae* may benefit host health by boosting *B. acidifaciens* abundance. Further metabolomic research targeting *B. acidifaciens*-derived beneficial metabolites should examplify the significance of the present study.

As *H. parainfluenzae* shapes the gut microbiota, the lack of ectopic gut colonization may mitigate the adverse effects associated with its ectopic colonization [50]. Ectopic colonization of oral bacteria in the gut is known to disrupt microbial balance, leading to immune dysregulation and metabolic disturbances [9,10,50,51]. The tailored dose used in this study appears to minimize the risk of ectopic colonization while still being sufficient to modulate microbiota and immune responses [14]. Whether a higher dose mimicking oral dysbiosis could promote ectopic colonization and trigger inflammatory dysbiosis remains to be clarified.

Without establishing ectopic colonization, *H. parainfluenzae* may influence microbial communities through transient interactions. As a core member of the human oral microbiota, it is continuously introduced into the gut, where it may influence microbial dynamics, particularly during periods of perturbation such as post-antibiotic reconstitution. While our findings suggest a potential modulatory role, further studies are needed to determine whether its depletion has long-term consequences for gut health.

The lack of a requirement for sustained *H. parainfluenzae* colonization at oral or gut sites suggests that viable microorganisms may not be necessary to exert modulatory effects. This highlights the potential of *H. parainfluenzae* as a postbiotic. Exploring the non-viable microorganism or its components holds scientific and commercial promise, while eliminating the risks associated with ectopic colonization and serious infection caused by the microorganism [52].

Several limitations exist in this study. As the research was limited to female NOD mice and a single *H. parainfluenzae* strain, it remains uncertain whether the findings can be generalized to other murine hosts or humans, and whether the observed effects are specific to this bacterial strain, species, or broader taxonomic groups. The long-term stability of *H. parainfluenzae*-induced microbial shifts requires clarification. Another limitation of the study is the lack of direct observation of mucosa-associated lymphoid tissues, which could provide further insights into systemic immunomodulation. Future research should clarify the mechanisms underlying gut microbiota regulation and its consequences in additional disease models, and validate these findings in human cohorts.

## Conclusions

This study demonstrates that oral *H. parainfluenzae* modulates post-antibiotic gut community reconstitution, marked by enrichment of *Bacteroides acidifaciens* and a reduction in splenic dendritic cell abundance, without ectopic gut colonization or the need for sustained oral colonization. These findings reinforce the oral–gut axis, indicating that commensal-derived signals can shape intestinal ecosystems and systemic immunity, and they suggest a plausible postbiotic mode of action.

Given the use of female NOD mice and a single *H. parainfluenzae* strain, further work should validate these effects across host backgrounds and strains, incorporate direct assessment of mucosa-associated lymphoid tissues, and elucidate metabolites and pathways—particularly those linked to *B. acidifaciens*. The use of inactive or non-viable bacteria, structural components, or outer membrane vesicles should also be explored. If confirmed, *H. parainfluenzae*-based postbiotics may offer a safe, non-colonizing strategy to support microbiota reconstitution after antibiotics and to mitigate downstream inflammatory sequelae.

## Supplementary Material

Supplementary material Supplementary Figure S1

## Data Availability

The sequencing data have been deposited in the SRA under BioProject ID PRJNA1245280 (https://www.ncbi.nlm.nih.gov/bioproject/PRJNA1245280). Flow cytometry raw data are available at https://drive.google.com/drive/folders/1gVhTqVDTRkT0w4mdGYSo5VwME8Z0ZLiT. Other supporting data are available upon reasonable request.
