# Supplementary material for: Distinct trajectory of gut microbiota driven by a human oral commensal: insights from a murine study
Source: J Oral Microbiol. 2025 Oct 24;17(1):2569524. doi: 10.1080/20002297.2025.2569524 (PMC12557821; doi:10.1080/20002297.2025.2569524)
Supplement: Supplementary material — Supplementary Figure S1 [file ZJOM_A_2569524_SM4759.docx]

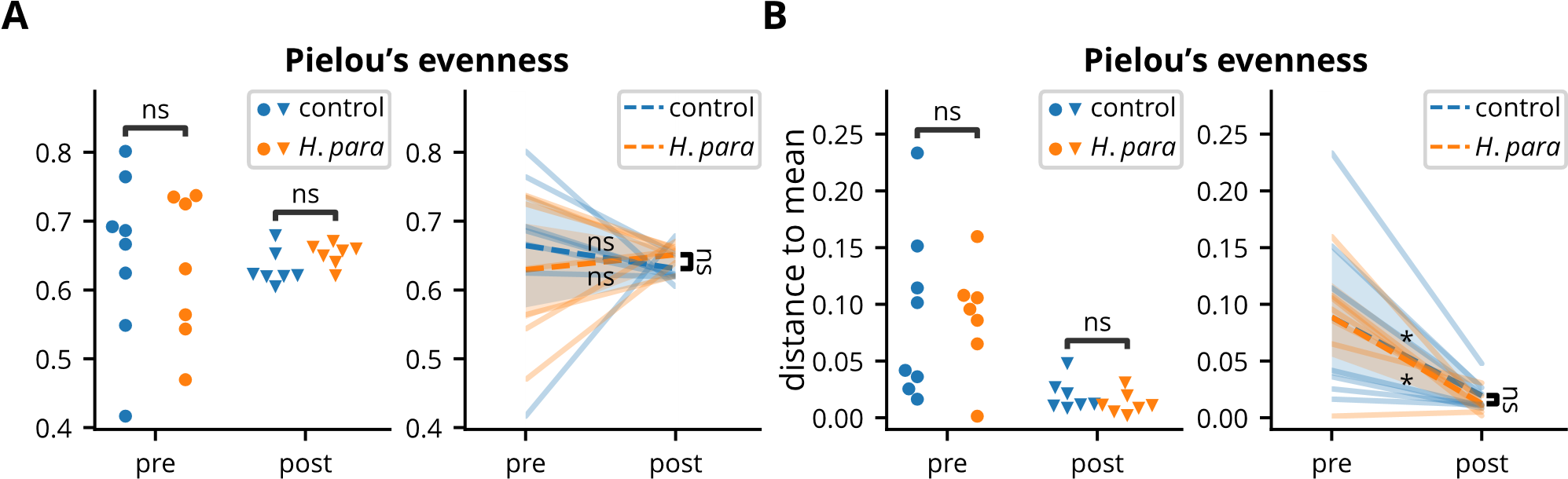


**Supplementary Figure 1. Analyses of microbial evenness**

1. Evenness comparing control and *H. parainfluenzae* groups before and after inoculation. Nonpaired and paired analyses.
2. Distance-to-mean analyses illustrating individual variation within each group. Non-paired andpaired analyses.

Statistical comparisons were performed using the Wilcoxon signed-rank test for paired analyses and the Mann-Whitney U test for between-group comparisons. Statistical significance: *p* < 0.05 (*) and non-significant (ns).
